# Chorioamnionitis and Patent Ductus Arteriosus: A Systematic Review and Meta-Analysis

**DOI:** 10.1371/journal.pone.0138114

**Published:** 2015-09-16

**Authors:** Hye Won Park, Yong-Sung Choi, Kyo Sun Kim, Soo-Nyung Kim

**Affiliations:** 1 Department of Pediatrics, Konkuk University Medical Center, Seoul, Korea; 2 Konkuk University School of Medicine, Seoul, Korea; 3 Department of Pediatrics, Kyung Hee University School of Medicine, Seoul, Korea; 4 Department of Obstetrics and Gynecology, Konkuk University Medical Center, Seoul, Korea; Hôpital Robert Debré, FRANCE

## Abstract

**Background:**

Chorioamnionitis has recently been reported as a risk factor for various neonatal diseases, including cerebral palsy, bronchopulmonary dysplasia, and necrotizing enterocolitis, but its effect on patent ductus arteriosus (PDA) is unclear. We performed a systematic review and meta-analysis to evaluate the effect of chorioamnionitis on PDA.

**Methods:**

We searched PubMed, EMBASE, Cochrane Library, and KoreaMed databases using the terms: “intrauterine infection” or “maternal infection” or “antenatal infection” or “chorioamnionitis” or “placenta inflammation” or “placenta pathology” or “neonatal outcome” or “neonatal morbidity” or “PDA or patent ductus arteriosus” or “ductus arteriosus,” and “prematurity” or “very low birth weight infant.” Studies were included if they were randomized controlled trials, case–control studies, or cohort studies that included information relating to chorioamnionitis and PDA.

**Results:**

Among 1,571 studies, a total of 23 studies (17,708 cases) were included in the meta-analysis to analyze the relationship between chorioamnionitis and PDA, except one study that only included PDA requiring surgical ligation. The association between chorioamnionitis and PDA was statistically significant (odds ratio [OR] 1.43; 95% confidence interval [CI] 1.19, 1.72; P < 0.0001). In subgroup analysis, clinical chorioamnionitis was not associated with PDA (OR 1.28; 95% CI 1.00, 1.64, 1.790; P = 0.05), whereas histologic chorioamnionitis (OR 1.54; 95% CI 1.10, 2.15; P = 0.01) and chorioamnionitis diagnosed from both clinical and histologic findings (OR 1.75; 95% CI 1.07, 2.86; P = 0.03) showed significant associations with PDA. Chorioamnionitis did not increase the risk of PDA requiring surgical ligation (OR 1.23; 95% CI 0.69, 2.17; P = 0.48), and antenatal steroid use reduced the risk of PDA (OR 0.62; 95% CI 0.42, 0.90; P = 0.01) after chorioamnionitis.

**Conclusions:**

The results from this meta-analysis support an association between maternal chorioamnionitis and PDA in offspring.

## Introduction

Chorioamnionitis is a risk factor for preterm birth, but the relationship between chorioamnionitis and neonatal morbidity or mortality remains controversial. However, several meta-analyses recently reported chorioamnionitis as a risk factor for various neonatal diseases, such as cerebral palsy [1], retinopathy of prematurity [2], bronchopulmonary dysplasia [3], and necrotizing enterocolitis [4]. Although the routine treatment of patent ductus arteriosus (PDA) is not recommended, persistent shunting through symptomatic PDA could increase the risk of neonatal mortality and morbidity, including chronic lung disease, intraventricular hemorrhage, and necrotizing enterocolitis [5, 6]. In the case of PDA, it has been reported that chorioamnionitis was a risk factor for unresponsiveness to medical therapy with cyclooxygenase inhibitors [7, 8]. The effect of antenatal steroids after chorioamnionitis has been reported [9], but the effect of chorioamnionitis itself on PDA occurrence is unclear. We performed a systematic review and meta-analysis to evaluate the effect of maternal chorioamnionitis on PDA in offspring.

## Materials and Methods

### Search Strategy and Study Selection

We performed this meta-analysis in accordance with the PRISMA guidelines developed for systematic reviews and meta-analyses (S1 PRISMA Checklist). We searched PubMed, EMBASE, Cochrane Library, and KoreaMed databases using the terms: “intrauterine infection” or “maternal infection” or “antenatal infection” or “chorioamnionitis” or “placenta inflammation” or “placenta pathology” or “neonatal outcome” or “neonatal morbidity” or “PDA” or “patent ductus arteriosus” or “ductus arteriosus,” and “prematurity” or “very low birth weight infant.” Manual searches were also performed on the reference lists of included studies and other electronic databases. No restrictions were applied on language. The last search was performed on September 19, 2014. The titles and abstracts of the articles were initially screened, and the full-text articles were independently reviewed by two reviewers (HW Park and YS Choi) using the selection criteria to determine inclusion in the meta-analysis.

Studies were included for meta-analysis if they met the following criteria: 1) study design: randomized controlled trial, case-control study, or prospectively or retrospectively matched cohort study; 2) patients: preterm infants who were born to a mother for whom information about chorioamnionitis was available; 3) intervention: clinical or histologic chorioamnionitis; and 4) outcomes: PDA (medically treated, surgically treated, or clinically diagnosed). Studies were excluded from the meta-analysis if they were case reports, case series, or single-arm cohort studies.

### Data Extraction and Study Quality Assessment

Two authors (HW Park and YS Choi) independently reviewed the full text of all included studies and extracted data with a data extraction form. We extracted the following data: first author, publication year, study design, study location, study period, study population, definition of chorioamnionitis, definition of PDA, and incidence of PDA. We also extracted effect estimates, odds ratios for the case–control studies, or relative risk for the cohort studies, along with the corresponding confidence interval, from each of the studies.

Disagreements were resolved through interpretation by discussion with a third reviewer (KS Kim). Methodological quality was independently assessed by two authors (HW Park and YS Choi) using the Newcastle–Ottawa Scale for all included studies. This scale assesses study quality by evaluating three domains: selection (four items), comparability (one item), and outcome/exposure (three items) for cohort studies and case-control studies, respectively. A study that meets the criteria can be awarded a star, except for the comparability domain (which has a maximum of two stars). The overall score is the total number of stars given, with a maximum of 9. Overall scores of 0–4, 5–7, and 8–9 stars correspond to studies of low, moderate, and high quality. Any disagreements between authors were resolved by discussion.

### Data Synthesis and Statistical Analysis

We conducted a meta-analysis to generate pooled estimates for the association between maternal chorioamnionitis and PDA in offspring using the RevMan 5.3.5 software (Cochrane Library; http://tech.cochrane.org/revman/download) and Comprehensive Meta-Analysis Version 3 (Biostat, Englewood, NJ, USA).

Statistical heterogeneity was assessed using the I^2^ statistic (percentage of total variation across studies), and I^2^ >50% was used to detect significant heterogeneity. If there was no heterogeneity, the fixed effects model was used for the meta-analysis. A random effects model was used for analysis if there was substantial statistical heterogeneity. A sensitivity analysis was conducted to evaluate the robustness of the conclusion by removing each study sequentially and to examine the effect of the elimination of each study on the pooled odds ratio (OR) results. A cumulative analysis was performed to detect temporal trends by adding one study at a time according to the year of publication.

Publication bias was assessed by the Begg and Mazumdar rank correlation test, the Egger’s regression test, and inspection of the funnel plot. A funnel plot and the Egger’s regression test were used to detect asymmetry based on the distribution of effect sizes against the standard errors.

## Results

### Characteristics of the Included Studies

**Fig 1:**
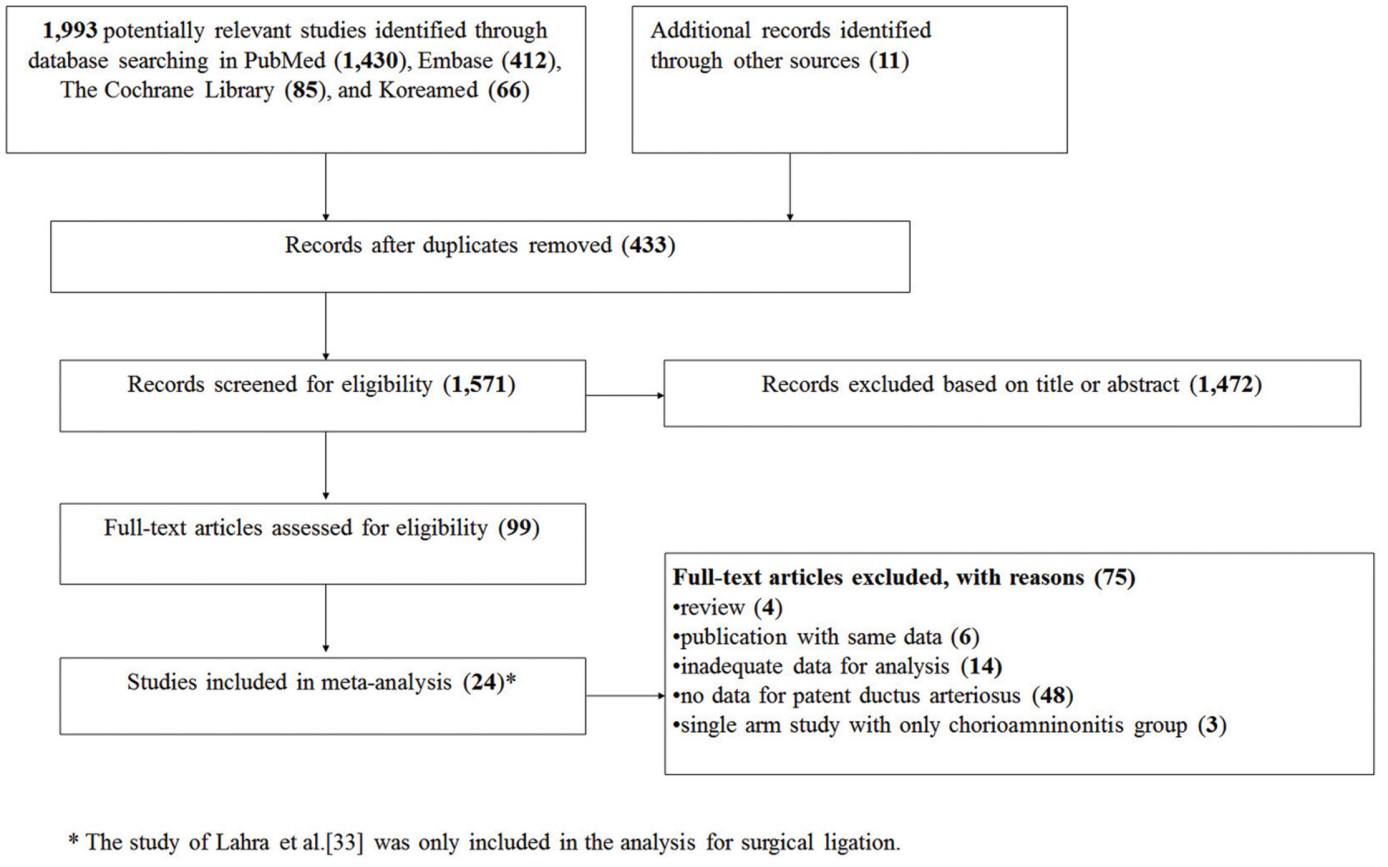
Flow diagram of literature selection. * The study by Lahra et al.[5] was only included in the analysis for surgical ligation.

A total of 1,993 potentially relevant studies were identified from database searching, and 1,970 of them were excluded for the reasons listed in Fig 1. After a complete survey of the literature, we included 23 studies (21 cohort and 2 case-control studies) in the systematic review. The 23 eligible studies included in the meta-analysis [10–32] examined a total of 17,708 mothers, of whom 4,681 had chorioamnionitis and 13,027 did not.

The quality assessment of the included studies was performed using the Newcastle-Ottawa Scale, and the results are presented in Table 1.

**Table 1:** Characteristics of studies included in this meta-analysis.

Study	Location	Study design	Population	Diagnosis, definition of PDA	Quality score*
**Histologic and clinical CA**					
Arayici 2014	Turkey	Retrospective cohort	GA ≤32 weeks and/or birth weight ≤ 1,500 g	Echocardiography, size >1.5 mm, LA/aorta >1.5 and/or left-to-right shunting, end diastolic reversal of blood flow in aorta	8
Been 2009	The Netherlands	Prospective cohort	GA ≤ 32 weeks	Echocardiography	7
Erdemir 2013	Turkey	Prospective cohort	GA < 35 weeks	NA	6
Mu 2008	Taiwan	Prospective cohort	birth weight ≤ 1,500 g	Echocardiography, and clinical signs of PDA	7
Ogunyemi 2003	USA	Retrospective cohort	GA, 24–32 weeks	NA	7
Seliga-Siwecka 2013	Poland	Prospective cohort	GA < 32 weeks	Echocardiography	6
**Clinical CA**					
Barrera-Reyes 2011	Mexico	Prospective cohort	GA < 34 weeks and birth weight < 1,500 g	NA	8
Botet 2010	Spain	Case–control	birth weight < 1,500 g	Presence of PDA (including surgical ligation)	8
Garcia-Munoz 2014	Spain	Prospective cohort	birth weight < 1,500 g	Echocardiography, requiring treatment (medical or surgical)	7
Soraisham 2009	Canada	Prospective cohort	GA < 33 weeks	Clinical signs and requiring treatment (medical or surgical)	7
**Histologic CA**					
Ahn 2012	Korea	Prospective cohort	GA < 34 weeks	Echocardiography, requiring treatment (medical or surgical)	8
De Felice 2005	Italy	Prospective cohort	Birth weight ≤ 1,500 g	NA	6
Dessardo 2012	Croatia	Prospective cohort	GA < 32 weeks	Echocardiography, and clinical signs of PDA	7
Ecevit 2014	Turkey	Retrospective cohort	GA < 37 weeks	NA	6
Elimian 2000	USA	Retrospective cohort	Birth weight, 500–1,750 g	PDA requiring treatment (medical or surgical)	7
Hendson 2011	Canada	Prospective cohort	GA ≤ 32 weeks and birth weight ≤ 1,250 g	Clinical diagnosis or radiologic diagnosis	6
Liu 2014	China	Prospective cohort	GA ≤ 34 weeks	Echocardiography, and clinical symptoms of PDA	7
Perrone 2012	Italy	Prospective cohort	GA, 23–31 weeks	Echocardiography, size >1.4 mm/kg body weight, LA/aorta >1.4, LA enlargement, shunting, end diastolic reversal of blood flow in descending aorta	6
Prendergast 2011	UK	Prospective cohort	GA ≤ 32 weeks	Requiring treatment (medical or surgical)	5
Rocha 2006	Portugal	Retrospective cohort	GA ≤ 34 weeks	Echocardiography	8
Sato 2011	Japan	Retrospective cohort	GA < 30 weeks	Hemodynamically significant PDA and requiring medical treatment	7
Schlapbach 2010	Switzerland	Case–control	GA, 25–32 weeks	NA	8
Tsiartas 2013	Czech Republic	Prospective cohort	GA: 24–36 weeks	NA	7
Lahra 2009 ^†^	Australia	Retrospective cohort	GA < 30 weeks	Echocardiography, requiring surgical treatment	8

*The quality assessment of studies was performed using the Newcastle-Ottawa Scale.

^**†**^ This study was only included in meta-analysis for the relationship between chorioamnionitis and surgical ligation.

**Abbreviation;** GA: gestational age at birth, LA: left atrium, PDA: patent ductus arteriosus, CA: chorioamnionitis

Six studies defined chorioamnionitis based on both clinical and histologic findings [15, 18, 19, 27, 28, 30]. In four studies[10–13], chorioamnionitis was diagnosed based on clinical findings, and the remaining 14 studies diagnosed it only on the basis of histologic findings of the placenta (one of these was the study that only included surgical ligation [33]) [14, 16, 17, 20–26, 29, 31–33]. In cases of clinical chorioamnionitis, criteria that were used in most studies were similar: the Gibbs criteria for chorioamnionitis [34], on the basis of maternal fever and two or more of the following additional criteria: maternal tachycardia, fetal tachycardia, uterine tenderness, foul odor of the amniotic fluid, and maternal leukocytosis. There were small variations in diagnostic criteria, such as fever (≥ 38 or 38.3°C), maternal leukocytosis (> 12,000/mm^3^ or 15,000/mm^3^ or 18,000/mm^3^), and fetal tachycardia (heart rate > 160/min or 180/min). In three studies, the elevation of C-reactive protein was included in the diagnostic criteria [11, 19, 20]. Histologic chorioamnionitis was commonly diagnosed based on the infiltration of polymorphonuclear leukocytes in the placental membranes, including the Salfia criteria [35], Blanc criteria [36], and criteria of the Amniotic Fluid Infection Nosology Committee [37].

**Fig 2:**
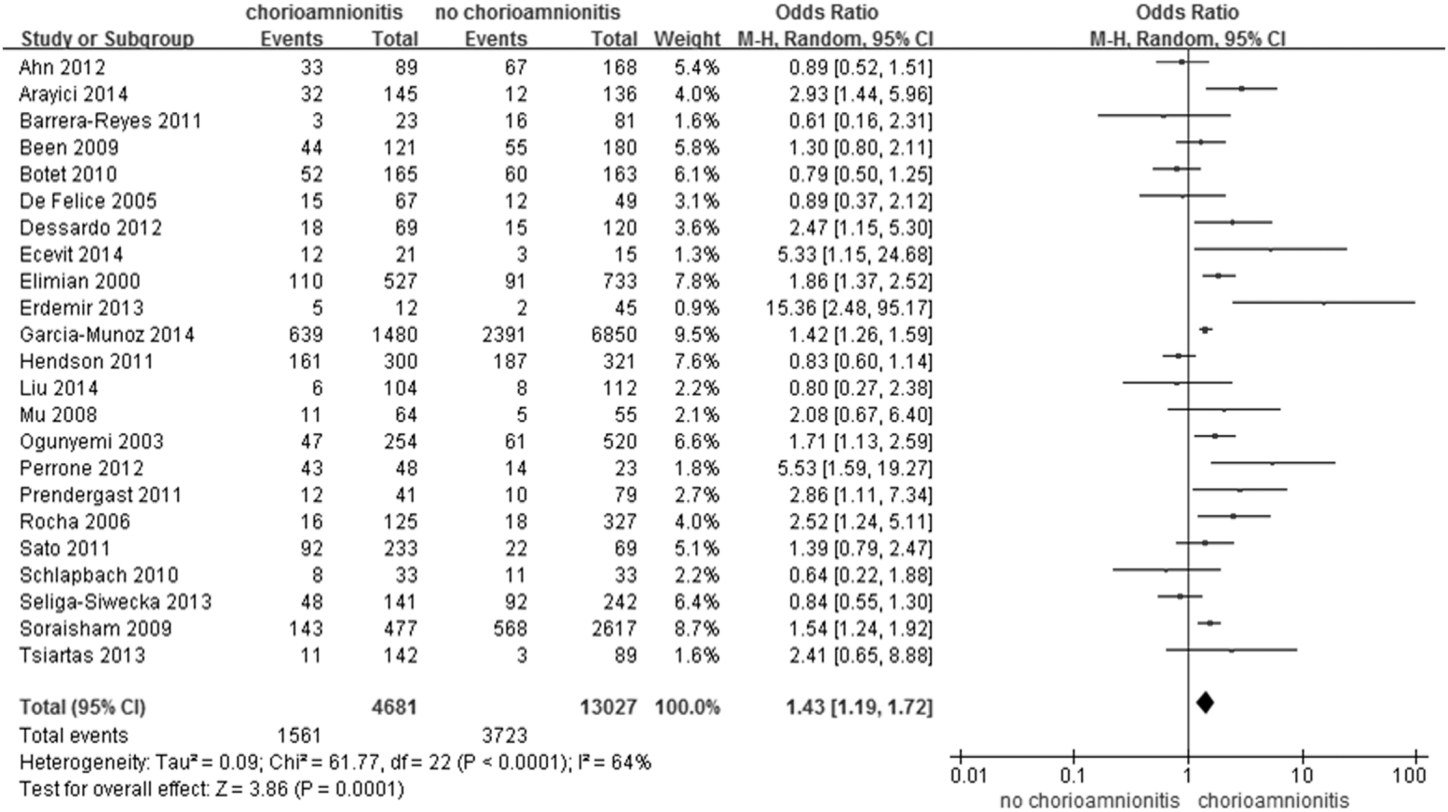
Meta-analysis for the relationship between maternal chorioamnionitis and neonatal patent ductus arteriosus.

**Fig 3:**
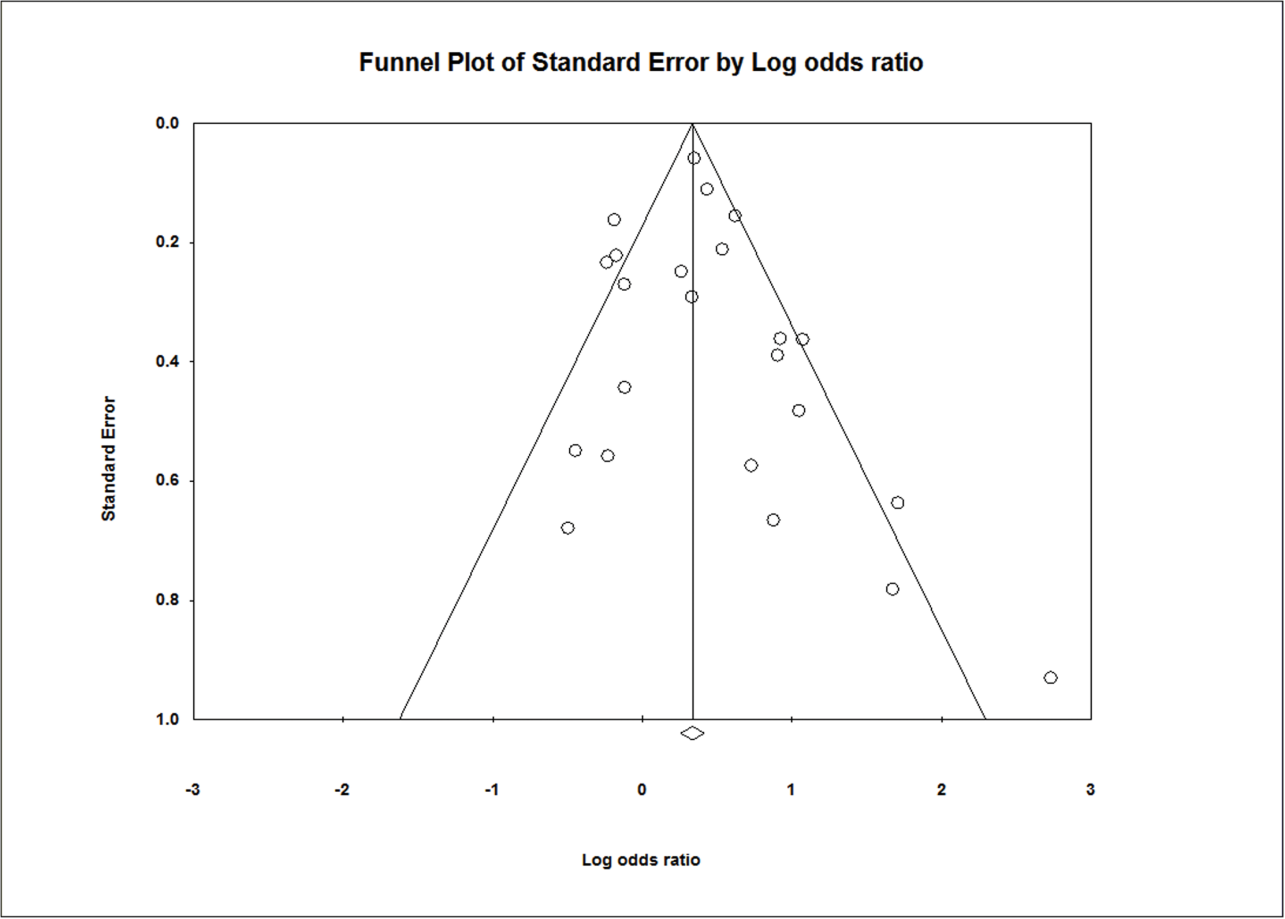
Funnel plot of the publications and meta-analysis evaluating the effect of chorioamnionitis on patent ductus arteriosus.

As a primary outcome, PDA was diagnosed in 5,284 of a total of 17,708 (30%) infants, including 1,561 of 4,681 (33.3%) infants of a mother with chorioamnionitis and 3,723 of 13,027 (28.6%) infants of a mother without chorioamnionitis. Overall chorioamnionitis, including that diagnosed based on clinical or histologic findings, was significantly associated with PDA (OR 1.43; 95% CI 1.19, 1.72; *P* <0.0001; Fig 2). The results of the heterogeneity assessment of overall studies (*P* < 0.0001; I^2^ = 64%) indicate the presence of heterogeneity across the studies. Thus, we used a random effects model for meta-analysis. We found no statistical evidence of publication bias in the studies from the results of the Begg-Mazumdar rank correlation test (*P* = 0.13) and Egger’s regression test (*P* = 0.48), as well as inspection of the funnel plot (Fig 3). A sensitivity analysis revealed no significant change in the pooled results caused by the sequential exclusion of individual studies (S1 Fig), and the results of a cumulative analysis also provided similar results over time (S2 Fig).

**Fig 4:**
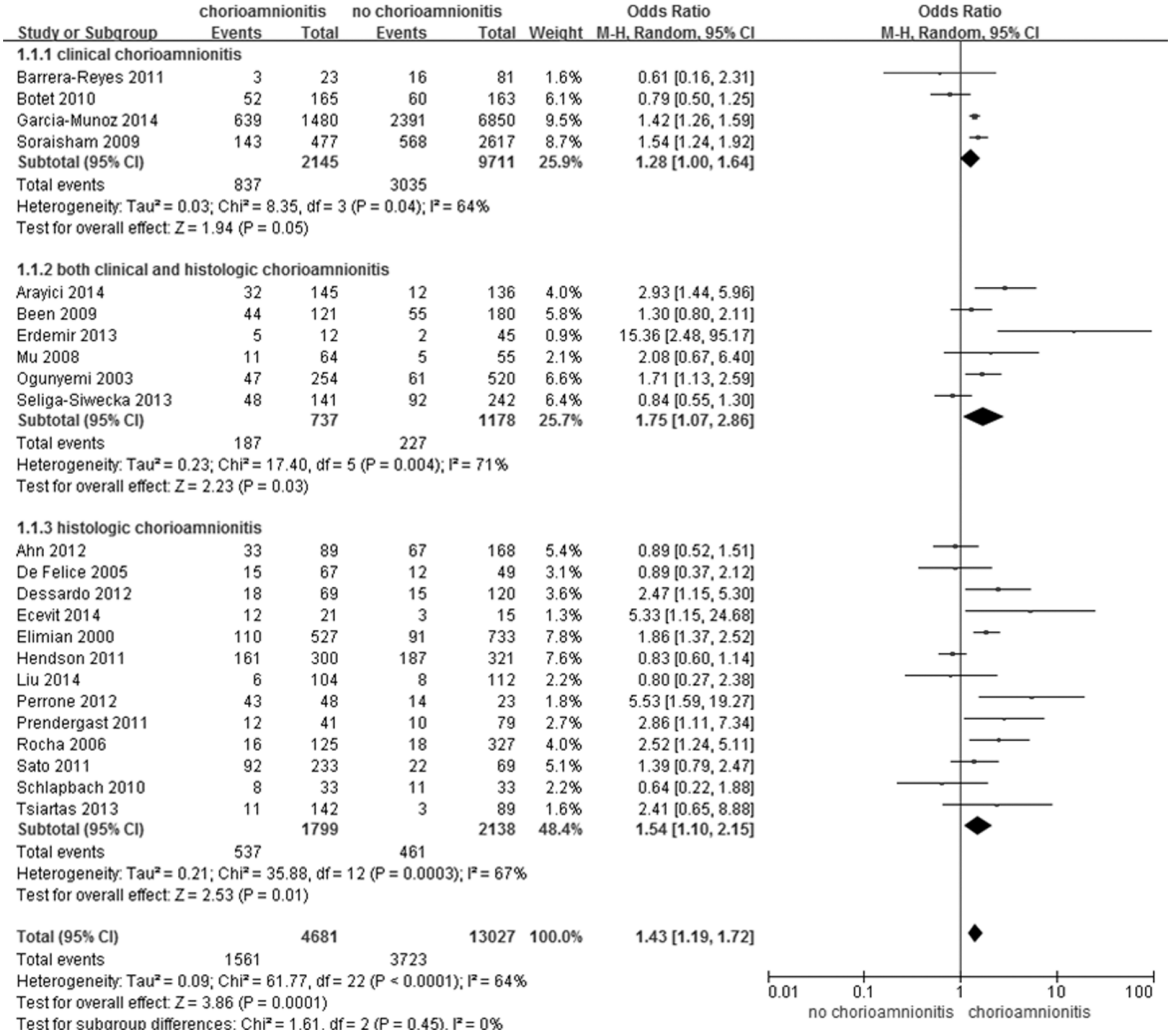
Subgroup-analysis according to diagnosis of both types of chorioamnionitis for neonatal patent ductus arteriosus.

In the subgroup analysis according to type of chorioamnionitis (clinical or histologic or both), the incidences of PDA were 39.0% vs 31.3% in patients with or without clinical chorioamnionitis, 29.8% vs 21.6% in patients with or without histologic chorioamnionitis, and 25.4% vs 19.3% in patients with or without both types of chorioamnionitis. In subgroup analysis according to types of chorioamnionitis, clinical chorioamnionitis was not associated with PDA (OR 1.28; 95% CI 1.00, 1.64, 1.790; *P* = 0.05; Fig 4). However, both groups (clinical and histologic chorioamnionitis) (OR 1.75; 95% CI 1.07, 2.86; *P* = 0.03) and histologic chorioamnionitis only (OR 1.54; 95% CI 1.10, 2.15; *P* = 0.01), including histologic examination in the diagnosis of chorioamnionitis, showed significant associations with PDA (Fig 4).

In the subgroup analysis according to study design (prospective and retrospective, including case–control studies), chorioamnionitis was associated with PDA in both groups (OR 1.346; 95% CI 1.1061, 1.708; *P* = 0.014 in the prospective studies, and OR 1.440; 95% CI 1.192, 1.739; *P* < 0.0001 in the retrospective studies; S3 Fig).

The presence of a fetal inflammatory response was not significantly associated with PDA (OR 0.57; 95% CI 0.13, 2.5; *P* = 0.45).

**Fig 5:**
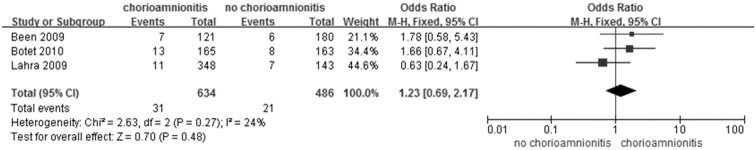
Meta-analysis for the relationship between maternal chorioamnionitis and neonatal patent ductus arteriosus requiring surgical ligation.

The incidence of PDA requiring surgical ligation did not differ between the chorioamnionitis group and non-chorioamnionitis group (OR 1.23; 95% CI 0.69, 2.17; *P* = 0.48; Fig 5). We used a fixed effects model because the heterogeneity test showed no evidence of heterogeneity between the included studies (*P* = 0.27; I^2^ = 24%). There was no statistical evidence of publication bias; the Begg-Mazumdar rank correlation test (*P* = 1.0) and Egger’s regression test (*P* = 0.88) did not suggest the presence of publication bias in this analysis.

**Fig 6:**
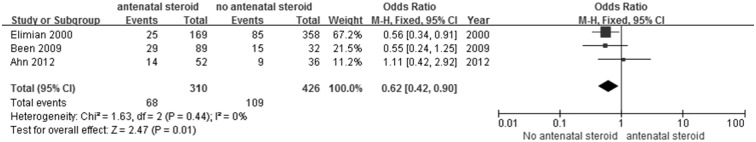
Meta-analysis for the relationship between antenatal steroid and neonatal patent ductus arteriosus after maternal chorioamnionitis.

Among patients with chorioamnionitis, administration of antenatal steroid was associated with a decreased risk of PDA compared to the non-use of antenatal steroid (OR 0.62; 95% CI 0.42, 0.90; *P* = 0.01; Fig 6). Inspection of the funnel plot was not sufficient to rule out publication bias because only three studies were included in this analysis but the Begg–Mazumdar rank correlation test (*P* = 0.30) and Egger’s regression test (*P* = 0.50) did not suggest the presence of publication bias. Because there was no evidence of heterogeneity between the included studies (*P* = 0.44; I^2^ = 0.0001%), we used a fixed effects model for meta-analysis.

## Discussion

The incidence of PDA has been reported in 55% of extremely low birth weight infants (birth weight < 1000 g), and 60–70% of preterm infants who were born at 28 weeks of gestation or earlier compared to the incidence in term infants (57/100,000 live births) [6]. The patency of ductus arteriosus is regulated by the balance between vasodilating and vasoconstricting factors [38]. Increased sensitivity to vasodilatory material (NO or prostaglandins) and decreased sensitivity to oxygen to constrict the ductus could explain the higher incidence of PDA in preterm than term babies. In the lamb model, the sensitivity of the ductus to nitric oxide (NO; a vasodilator) depends on gestational age; the ductus showed a tendency toward more pronounced NO-mediated relaxation during early gestation (corresponding to approximately 29 weeks in humans) compared with late gestation (corresponding to more than 34 weeks in humans) [39, 40]. Preterm infants also have a persistent response to prostaglandins compared with full term babies because the decrease in the PGE_2_ receptor has not occurred in preterm infants [38].

In this meta-analysis, chorioamnionitis showed a statistically significant association with PDA (OR 1.43; 95% CI 1.19, 1.72; *P* <0.0001; Fig 2) when diagnosed by histological examination of the placenta (OR 1.75; 95% CI 1.07, 2.86; *P* = 0.03 in both groups and OR 1.54; 95% CI 1.10, 2.15; *P* = 0.01 in histologic chorioamnionitis; Fig 4). The role of infection was also considered in maintaining the patency of ductus arteriosus. In rats, relaxation of the ductus was observed after an injection of lipopolysaccharide via induction the production of inducible nitric oxide synthetase (iNOS) [41]. Infection could induce production of iNOS and cyclooxygenase (COX)-2, resulting in the increased production of vasodilatory prostaglandins as well as constituting NOS and COX-1, which are expressed under normal conditions [39]. Kim et al. [7] demonstrated increased COX-1 expression in the umbilical artery in infants with intrauterine infection compared to those without intrauterine infection. Thus, an infant born to a mother with chorioamnionitis could have a persistently opened ductus arteriosus due to increased NO and vasodilatory prostaglandins. In addition to prenatal infections such as chorioamnionitis, postnatal infections are also associated with PDA. Gonzalez et al. [42] reported increased risks due to failed PDA closure, PDA recurrence, and increased levels of a prostaglandin (6-ketoprostaglandin F_1α_ [6-keto PGF_1α_]) and tumor necrosis factor alpha in infants with postnatal infections. Ductal vasodilator prostaglandin, 6-keto PGF_1α_, increased in postnatal infection [42] but not in intrauterine inflammation [7].

Although chorioamnionitis was associated with PDA in the total group with PDA (OR 1.43; 95% CI 1.19, 1.72; *P* <0.0001), clinical chorioamnionitis did not show an association with PDA (OR 1.28; 95% CI 1.00, 1.64, 1.790; *P* = 0.05) in subgroup analysis. Clinical chorioamnionitis is diagnosed using only clinical findings. These are often particularly vague, and any diagnosis is thus highly subjective [43]. Pappas et al. [44] reported that 2.7% of cases of clinical chorioamnionitis showed normal findings on histologic examination of the placenta. This group, which was diagnosed on the basis of both clinical and histologic findings (OR 1.75; 95% CI 1.07, 2.86; *P* = 0.03), showed a slightly higher OR than those with histologic chorioamnionitis only (OR 1.54; 95% CI 1.10, 2.15; *P* = 0.01).

The presence of chorioamnionitis was significantly associated with unresponsiveness following the use of COX inhibitors [7, 8]; surgical ligation is an indication that the PDA has been unresponsive to medical treatment. In our meta-analysis of populations with or without chorioamnionitis, PDA requiring surgical ligation was not associated with chorioamnionitis (OR 1.23; 95% CI 0.69–2.17; *P* = 0.48, Fig 5). Various postnatal factors, including infection and the need for fluid management, in addition to chorioamnionitis, may affect PDA outcomes [42, 45]. In addition, the number of studies included in the analysis was small.

Been et al. [9] previously reported the effects of antenatal steroids on PDA after chorioamnionitis [9]. Our study also showed a decreased risk of PDA with the use of an antenatal steroid in chorioamnionitis (OR 0.62; 95% CI 0.42, 0.90; *P* = 0.01, Fig 6), as did another study [24] in addition to the results of Been’s study. Although there has been concern about the use of steroids in chorioamnionitis, it could be safe and effective even in women with premature rupture of the membrane based on several studies [9, 46–48]. Intrinsic tone of the ductus is lower in preterm infants who were born earlier than 28 weeks of gestation with increased sensitivity to vasodilators leading to failure of ductus constriction [38, 49][2,3][2,3]. Administration of corticosteroids reduces the sensitivity of the ductus to prostaglandin E_2_ [38] and inhibits the induction of iNOS and COX-2 in the presence of inflammation [39]. Moreover, the extreme preterm infant commonly has relative adrenal insufficiency because the adrenal cortex cannot produce cortisol sufficiently until 23–30 weeks of gestation [50]. Thus, the use of an antenatal corticosteroid, especially in extreme preterm infants, could help to reduce the occurrence of PDA in the offspring of mothers with chorioamnionitis. The administration of a postnatal corticosteroid also indicated the effect on the decrease in the incidence of PDA [51, 52]. No statistical association was found between the presence of a fetal inflammatory response and PDA (OR 0.57; 95% CI 0.13, 2.5; *P* = 0.45); however, only three studies [19, 25, 32] were included, and there was substantial heterogeneity among studies (*P* = 0.02; I^2^ = 84%).

PDA is a multi-factorial disease that occurs during the neonatal period. We conducted a meta-regression analysis using a random effects model to assess the impact of gestational age on the occurrence of PDA as a covariate. A meta-regression using the length of gestation (both the maximal value and the mean value) as a covariate provides *P*-values as follows: *P* = 0.230 for the maximal gestational age (S4 Fig) and *P* = 0.944 for the mean gestational age (S5 Fig), thus indicating no association of the covariate with the log odds ratio (size of the choroamnionitis effect on PDA).

There are some limitations to be addressed regarding the present study. First, observational study designs are prone to both bias and confounding, which exclusively affect non-randomized studies [4]. However, in the subgroup analysis according to study design (prospective and retrospective, including case–control studies), there was no significant difference in the OR between the studies according to study design (OR 1.346; 95% CI 1.1061, 1.708; *P* = 0.014 in the prospective studies, and OR 1.440; 95% CI 1.192, 1.739; *P* < 0.0001 in the retrospective studies; S3 Fig). Moreover, the Newcastle–Ottawa Scale scores indicated moderate to high quality for the methodology used in the included studies. Second, PDA was diagnosed in 30% and 33.3% of infants by virtue of the presence of chorioamnionitis in this study. However, the actual incidences of PDA in the study populations are unclear; 10 studies [10, 12, 15, 16, 20–22, 27, 29, 31] did not state whether PDA was diagnosed echocardiographically.

This systematic review and meta-analysis demonstrated an association between maternal chorioamnionitis and PDA in offspring, especially in histologically diagnosed chorioamnionitis. When routine treatment is not recommended and medical or surgical treatment is associated with potential risks [5], the presence of chorioamnionitis may predict prolonged PDA and a need for early treatment. Chorioamnionitis did not exhibit any association with PDA that required surgical ligation, but the prescription of antenatal steroids reduced the risk of PDA development after chorioamnionitis. Well-designed randomized controlled trials are needed to adjust for other postnatal factors affecting PDA outcomes. It would then be possible to evaluate the effect of chorioamnionitis alone, and thus the need for PDA treatment.

## Supporting Information

S1 Fig Sensitivity analyses of the relationship between maternal chorioamnionitis and neonatal patent ductus arteriosus. Abbreviations: CI, confidence interval; CA, chorioamnionitis.
(TIF)

S2 Fig Cumulative analyses of the relationship between maternal chorioamnionitis and neonatal patent ductus arteriosus. Abbreviations: CI, confidence interval; CA, chorioamnionitis.
(TIF)

S3 Fig Subgroup analysis according to study design. (TIF)

S4 Fig Relationship between the gestational age (maximal value in inclusion criteria in each study) and the odds ratios and PDA in a random meta-regression analysis. (TIF)

S5 Fig Relationship between the gestational age (mean value in each study) and the odds ratios and PDA in a random meta-regression analysis. (TIF)

S1 PRISMA Checklist The PRISMA checklist. (DOC)
