# Supplementary figures and images for: Chorioamnionitis and Patent Ductus Arteriosus: A Systematic Review and Meta-Analysis
Source: PLoS One. 2015 Sep 16;10(9):e0138114. doi: 10.1371/journal.pone.0138114 (PMC4574167; doi:10.1371/journal.pone.0138114)

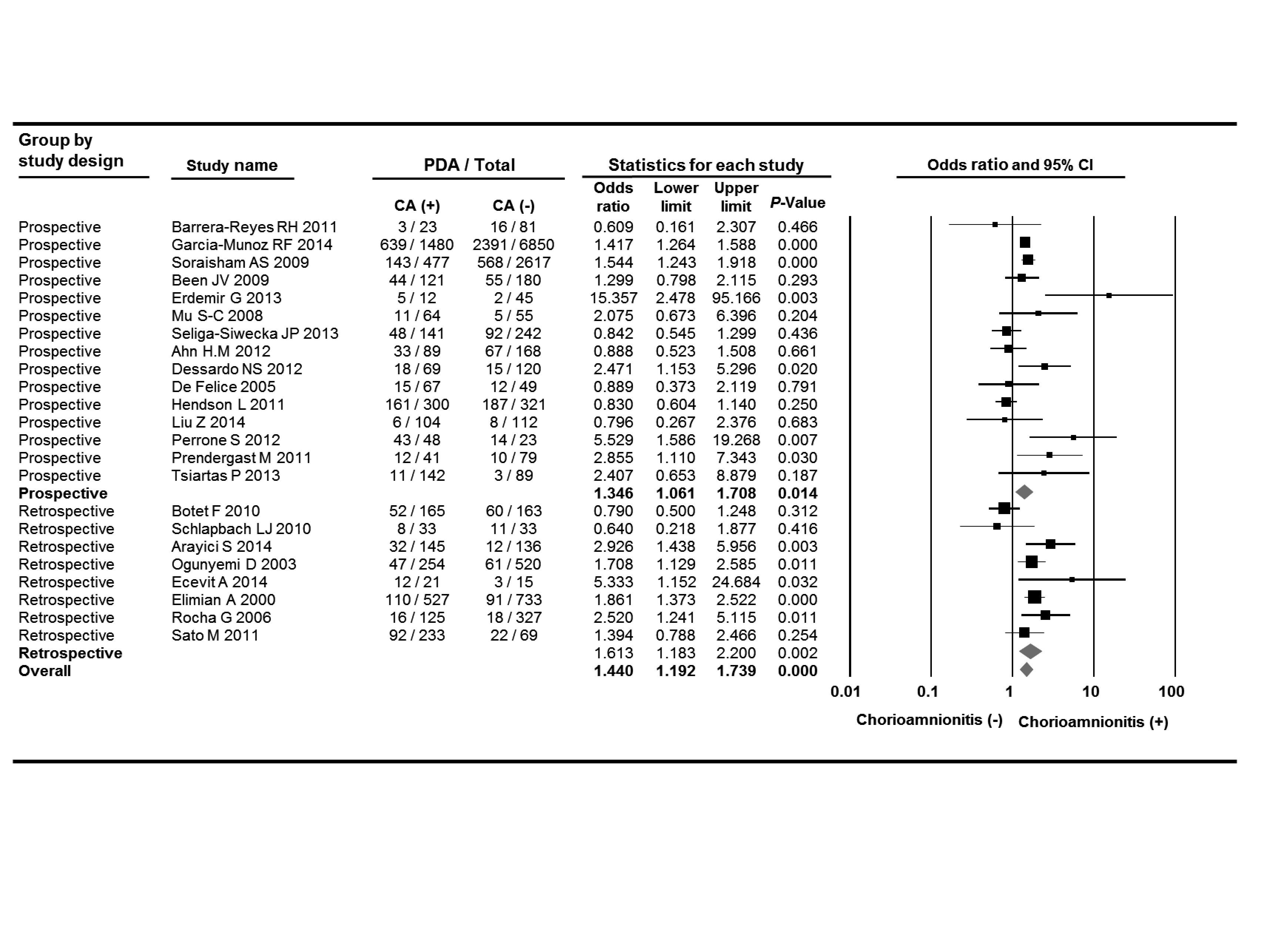

Supplement: S3 Fig — (TIF) [file pone.0138114.s003.tif]

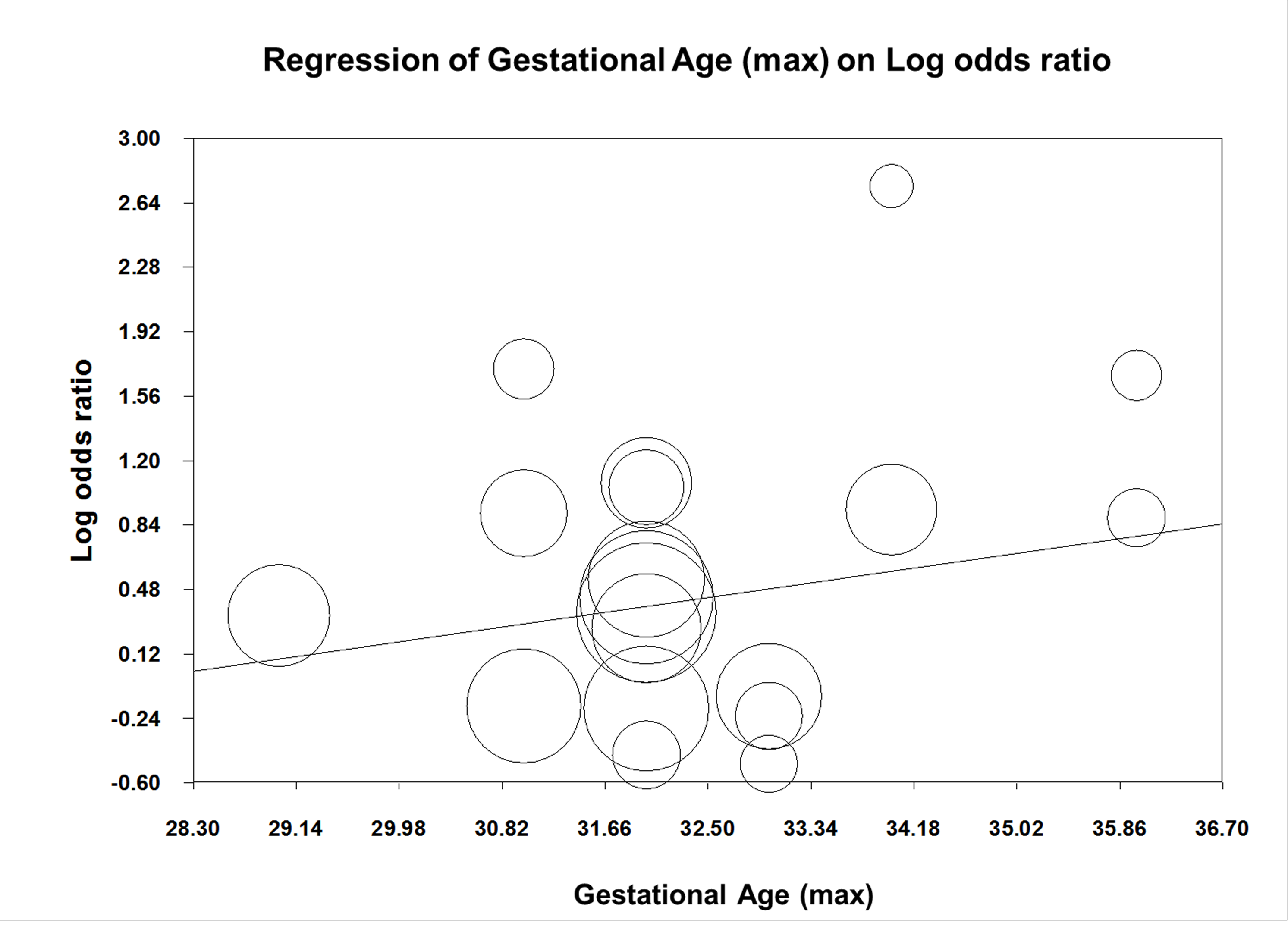

Supplement: S4 Fig — (TIF) [file pone.0138114.s004.tif]

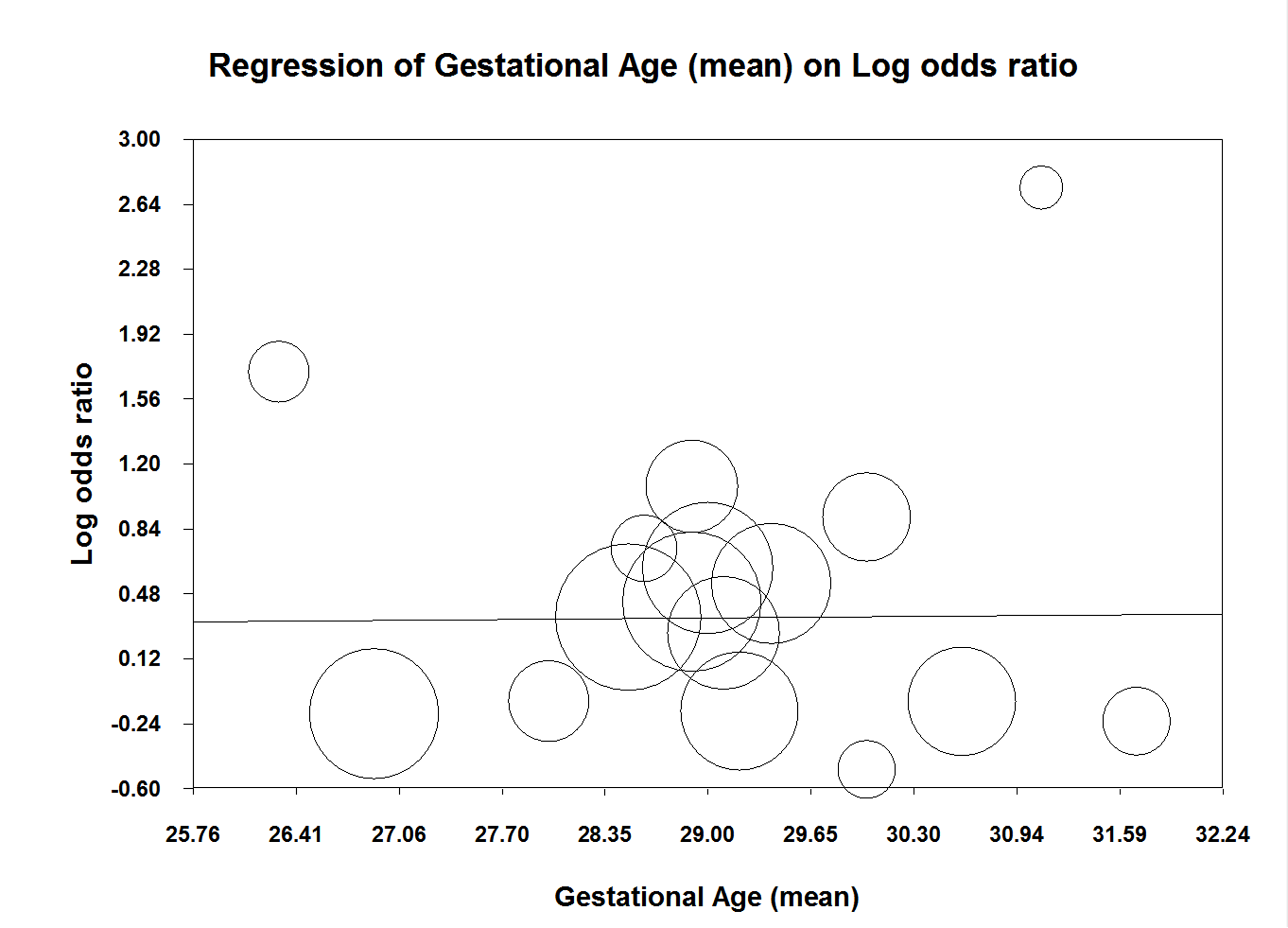

Supplement: S5 Fig — (TIF) [file pone.0138114.s005.tif]
